# A novel small-molecule MRCK inhibitor blocks cancer cell invasion

**DOI:** 10.1186/s12964-014-0054-x

**Published:** 2014-10-05

**Authors:** Mathieu Unbekandt, Daniel R Croft, Diane Crighton, Mokdad Mezna, Duncan McArthur, Patricia McConnell, Alexander W Schüttelkopf, Simone Belshaw, Andrew Pannifer, Mairi Sime, Justin Bower, Martin Drysdale, Michael F Olson

**Affiliations:** Molecular Cell Biology Laboratory, Cancer Resarch UK Beatson Institute, Garscube Estate, Switchback Road, Glasgow, G61 1BD UK; Drug Discovery Programme, Cancer Resarch UK Beatson Institute, Garscube Estate, Switchback Road, Glasgow, G61 1BD UK; Present address: European Screening Centre, Bo’Ness Road, Newhouse, ML1 5UH UK

## Abstract

**Background:**

The myotonic dystrophy kinase-related CDC42-binding kinases MRCKα and MRCKβ regulate actin-myosin contractility and have been implicated in cancer metastasis. Along with the related ROCK1 and ROCK2 kinases, the MRCK proteins initiate signalling events that lead to contractile force generation which powers cancer cell motility and invasion. A potential strategy for cancer therapy is to reduce metastasis by blocking MRCK activity, either alone or in combination with ROCK inhibition. However, to date no potent small molecule inhibitors have been developed with selectivity towards MRCK.

**Results:**

Screening a kinase-focused small molecule chemical library resulted in the identification of compounds with inhibitory activity towards MRCK. Medicinal chemistry combined with *in vitro* enzyme profiling led to the discovery of 4-chloro-1-(4-piperidyl)-N-[5-(2-pyridyl)-1H-pyrazol-4-yl]pyrazole-3-carboxamide (BDP00005290; abbreviated as BDP5290) as a potent MRCK inhibitor. X-ray crystallography of the MRCKβ kinase domain in complex with BDP5290 revealed how this ligand interacts with the nucleotide binding pocket. BDP5290 demonstrated marked selectivity for MRCKβ over ROCK1 or ROCK2 for inhibition of myosin II light chain (MLC) phosphorylation in cells. While BDP5290 was able to block MLC phosphorylation at both cytoplasmic actin stress fibres and peripheral cortical actin bundles, the ROCK selective inhibitor Y27632 primarily reduced MLC phosphorylation on stress fibres. BDP5290 was also more effective at reducing MDA-MB-231 breast cancer cell invasion through Matrigel than Y27632. Finally, the ability of human SCC12 squamous cell carcinoma cells to invade a three-dimensional collagen matrix was strongly inhibited by 2 μM BDP5290 but not the identical concentration of Y27632, despite equivalent inhibition of MLC phosphorylation.

**Conclusions:**

BDP5290 is a potent MRCK inhibitor with activity in cells, resulting in reduced MLC phosphorylation, cell motility and tumour cell invasion. The discovery of this compound will enable further investigations into the biological activities of MRCK proteins and their contributions to cancer progression.

## Background

Tumour cell invasion is a defining hallmark of malignancy [1]. For most types of solid tumours, patient mortality and much morbidity is attributable to metastatic disease, of which invasion is an obligatory component process. Current anticancer drugs mainly target tumour growth, and their clinical benefits at all stages of the disease typically are modest. By subduing cancer cell invasion, particularly in an adjuvant setting, molecularly-targeted inhibitors that blocked key invasion drivers would be expected to provide clinical benefit to a significant range of cancer patients with solid tumours at various stages.

Metastasis is a multi-step process powered by dynamic reorganization of the actin-myosin cytoskeleton and remodelling of the extracellular matrix, allowing cells to invade their local environment, cross tissue boundaries and spread *via* blood and lymphatic vessels to distal regions of the body [2]. Contraction of actin-myosin cytoskeletal structures generates the mechanical force required for cell motility and invasion [2]. A key element of the cytoskeletal contractile machinery is myosin II, which is regulated by phosphorylation of myosin II light chain proteins (MLC) at two key sites (Thr18 and Ser19) [3].

Members of the RhoGTPase family are central regulators of the actin-myosin cytoskeleton and have been shown to contribute to multiple processes associated with invasion and metastasis [2]. Cdc42 signals through effector proteins including the myotonic dystrophy kinase-related Cdc42-binding kinases α and β (MRCKα and MRCKβ), which are 190 kDa multi-domain proteins with ~80% amino acid identity across their kinase domains, that are expressed in a wide range of tissues [4]. MRCK and the Rho-regulated ROCK kinases belong to the AGC kinase family [5], and share ~45-50% amino acid identity in their N-terminal kinase domains, which is reflected in their shared abilities to phosphorylate a similar set of substrates including MLC and the inhibitory phosphorylation of the myosin binding subunit (MYPT1) of the MLC phosphatase complex [6]. However, MRCK and ROCK kinases may phosphorylate substrates, such as MLC, at different subcellular localizations due to their specific interactions with targeting proteins and/or lipids [7-10].

Importantly, it has been observed that the actin-myosin contractility required for the invasion of three-dimensional extracellular protein matrices by MDA-MB-231 breast cancer cells [6,11] and for the collective invasion of squamous cell carcinoma (SCC) cells through three dimensional collagen matrices in an organotypic model [12] were dependent on MRCK signalling. Elevated MRCKα expression was reported to contribute to Ras oncogene-driven SCC development in genetically-modified mice following repression of the Notch1 tumour suppressor [13]. In addition, gene expression analysis identified *MRCKα* as part of a breast cancer gene expression signature linked to poor patient prognosis and increased incidence of metastasis under five years [14]. These observations indicate that MRCK contributes to tumour cell invasiveness and may be an important driver of metastasis.

To date, there have been no potent and selective MRCK inhibitors reported that could be used to test the hypothesis that pharmacological inhibition of MRCK activity would reduce cancer cell invasion [4]. We now show that a 2-pyridyl pyrazole amide compound we discovered is a potent MRCK inhibitor with significant selectivity over the closely-related ROCK kinases both *in vitro* and in cells. Determination of the structure of the MRCKβ kinase domain associated with the compound reveals similarities to ADP in the way that it is associated with the nucleotide binding pocket. Treatment of MDA-MB-231 human breast cancer cells with BDP00005290 (BDP5290) was sufficient to fully inhibit MLC phosphorylation (pMLC) by targeting both cytoplasmic and peripheral actin-myosin bundles, in contrast to the ROCK selective inhibitor Y27632 that preferentially reduced cytoplasmic pMLC. Paralleling this observation, MDA-MB-231 cell invasion through Matrigel was more efficiently blocked by BDP5290 than Y27632. Invasion of SCC12 human squamous cell carcinoma into three-dimensional collagen matrices was strongly inhibited by BDP5290 but not Y27632, consistent with a previous report that MRCK, and not ROCK, was required for collective invasion by these tumour cells [12]. These results show that we have discovered a potent MRCK inhibitor that effectively blocks tumour cell invasion.

## Results and discussion

### MRCK inhibitor discovery

**Figure 1:**
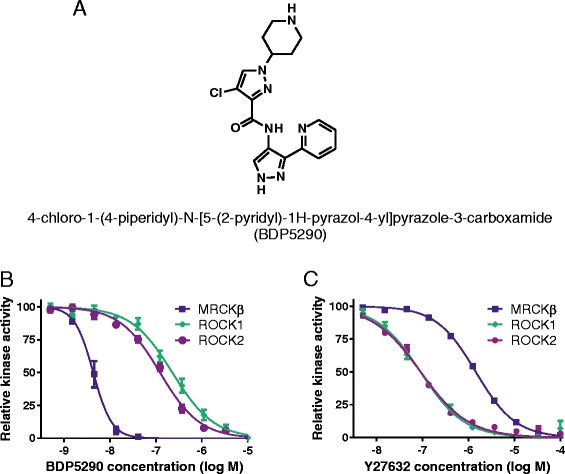
**Structure of BDP5290 and in vitro kinase inhibition profiles. (A)** Structure of 4-chloro-1-(4-piperidyl)-N-[5-(2-pyridyl)-1H-pyrazol-4-yl]pyrazole-3-carboxamide (BDP5290). **(B)** BDP5290 dose–response curves for inhibition of MRCKβ, ROCK1 and ROCK2 kinase activity *in vitro* at 1 μM ATP. **(C)** Y27632 dose–response curves for inhibition of MRCKβ, ROCK1 and ROCK2 kinase activity *in vitro* at 1 μM ATP. All results are shown as mean ± standard error of n ≥ 4 independent replicates.

After having established an *in vitro* fluorescence polarization-based MRCKβ kinase assay [11], a high-throughput screening campaign of 87,225 compounds was completed. There were 616 initial compound hits, which inhibited MRCKβ > 48% when screened at 30 μM (0.7% hit-rate). Of these, 492 compounds were selected for further evaluation and development. A screening cascade was designed to enable rapid and efficient identification of MRCK selective inhibitors. Iterative rounds of structure-activity relationship (SAR) chemistry on representatives of the initial screening hit matter, *in vitro* profiling and X-ray crystallography resulted in the discovery of 4-chloro-1-(4-piperidyl)-N-[5-(2-pyridyl)-1H-pyrazol-4-yl]pyrazole-3-carboxamide (BDP5290; Figure 1A) as an MRCK inhibitor with excellent potency and selectivity profiles. Synthesis route and methods for BDP5290 will be described in a subsequent manuscript (in preparation). Inhibitor dose–response assays at 1 μM ATP, which was close to the experimentally-determined K_m_ ATP values for the test kinases under the conditions of the enzyme assay (MRCKα, 0.9 μM; MRCKβ, 0.4 μM; ROCK1, 0.6 μM; ROCK2, 0.6 μM), revealed IC_50_ values of 17 nM for MRCKβ, 230 nM for ROCK1 and 123 nM for ROCK2 (Figure 1B). Ligand efficiency of BDP5290 was calculated to be 0.40 for MRCKβ [15]. Using the Cheng-Prusoff equation [16] and the experimentally-determined K_m_ ATP values, the calculated K_i_ values revealed 86 and 46-fold *in vitro* selectivity for MRCKβ over ROCK1 and ROCK2, respectively. The K_i_ of BDP5290 for MRCKα was 10 nM, which was slightly more than the K_i_ of 4 nM for MRCKβ. For comparison purposes, at 1 μM ATP the ROCK inhibitor Y27632 had *in vitro* IC_50_ values of 1.45 μM for MRCKβ and 91 nM for ROCK1 and ROCK2, which indicates lower on-target potency compared to BDP5290 and only 16-fold selectivity for ROCK kinases over MRCKβ (Figure 1C). Additional physicochemical properties of BDP5290 are listed in Table 1.

**Table 1:** **Properties of BDP5290**

Caco-2 Apical to Basolateral Efflux Ratio	233
Papp (× 10^−6^ cm s^−1^)	0.171
Polar surface area (Å^2^)	100.5
logD	0.786

**Figure 2:**
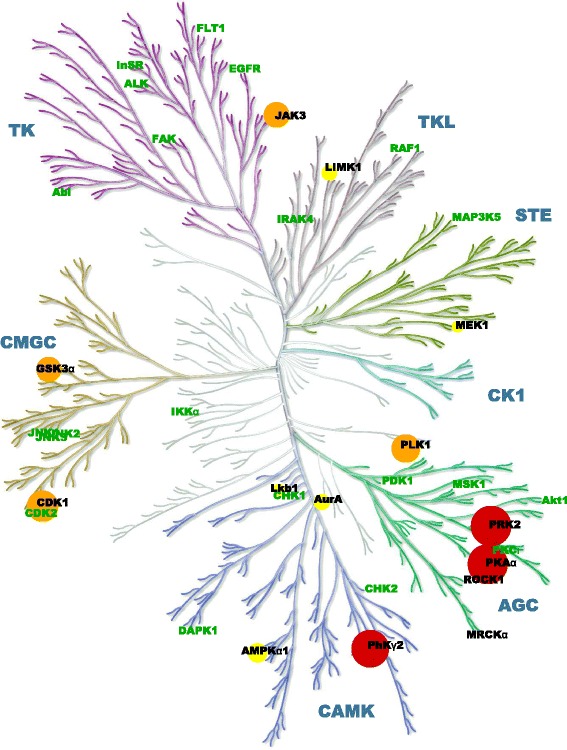
**Selectivity profile of BDP5290.** The percentage inhibition of 36 kinases by 10 μM BDP5290 was mapped onto the kinome phylogenetic tree. Kinases inhibited less than 25% have their names in green text. Kinases inhibited >25% and ≤50% have yellow circles, >50% and ≤75% have orange circles, and >75% have red circles. In each case, the size of the circle is proportional to the percentage inhibition. ROCK1 and MRCKα positions are indicated on the tree for orientation purposes. Illustration reproduced courtesy of Cell Signaling Technology, Inc. (www.cellsignal.com).

Kinase selectivity was determined by measuring the residual activity in the presence of 10 μM BDP5290 of a panel of 36 kinases. The results of this screening were mapped over the annotated human kinome phylogenetic tree [17] using Kinome Render [18] (Figure 2). Overall, the selectivity profile revealed that most kinases were unaffected by BDP5290 (green labels), with the AGC family kinases PRK2 and PKAα as well as the CAMK kinase PhKγ2 being the only ones inhibited >75% (red circles) at this concentration. These results show that BDP5290 is a potent MRCK inhibitor suitable as a chemical biology tool to characterize the contribution made by MRCK to cancer cell invasion.

### Structure of the MRCKβ-BDP5290 complex

**Figure 3:**
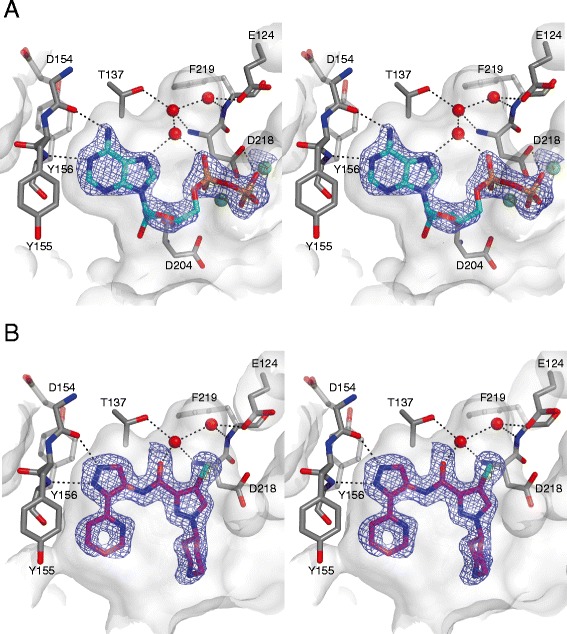
**Structure of MRCKβ in complex with ADP and BDP5290.** Stereo views of the MRCKβ active site in complex with ADP **(A)** or BDP5290 **(B)**. The ligands are shown in cyan and purple, respectively. Protein residues are coloured grey and labelled. Mg^2+^ ions are shown as light green spheres, selected water molecules are indicated by red spheres. Dotted black lines represent potential hydrogen bonding interactions. |*F*_o_|-|*F*_c_|, φ_c_ electron density maps calculated prior to the initial inclusion of the ligands in their respective models are shown contoured at 2.6σ. This figure was created using PyMOL.

To better understand ligand binding to MRCK, we determined the crystal structures of MRCKβ in complex with ADP to 1.73 Å, as well as with BDP5290 to 1.71 Å (Figure 3). Both structures adopted overall similar protein conformations to that we previously reported for MRCKβ [11], with RMSD values ≤ 1.25 Å after superposition on ≈ 380 Cα atoms.

The conformation of the MRCKβ kinase domain in complex with ADP resembled the previously reported complexes with fasudil or TPCA-1 [11], with the activation loop open and all important active site features ordered. The only significant difference in conformation around the active site is that the glycine-rich loop is moved out of the binding site by up to 4 Å to make space for the diphosphate moiety in the ADP complex. The nucleotide binds in the conformation typically observed for Ser/Thr protein kinases (Figure 3A). The adenine sits in a mostly hydrophobic pocket formed by the side chains of Ile82, Val90, Ala103, Tyr155, Tyr156, Leu207 and Phe370, while also making characteristic hydrogen bonding interactions with the hinge backbone (Asp154 carbonyl and Tyr156 amine). Additionally, the adenine N7 accepts a hydrogen bond from a water molecule that is part of a small buried water cluster (which we refer to as “pocket waters”) interacting with Glu124, Thr137, Asp218 and Phe219. The same water also hydrogen bonds to the α-phosphate of the nucleotide. The ribose moiety makes few directed interactions aside from a hydrogen bond to the backbone carbonyl of Asp204 (C-terminal of the catalytic loop) and a second hydrogen bond to a surface water that in turn also coordinates the diphosphate. The diphosphate is positioned by direct and indirect interactions with the side chains of Asn205 mediated by water molecules or stabilizing Mg^2+^ ions, as well as Asp218 of the DFG motif.

Bound BDP5290 (Figures 1A and 3B) fills a similar space in the MRCKβ active site as that occupied by either fasudil or TPCA-1 [11]. As a consequence, the glycine-rich loop adopts the closed conformation seen in the other inhibitor complexes. The pyridine-side pyrazole acts as the hinge binder, forming hydrogen bonds with the backbone of Asp154 and Tyr156. The pyridine moiety extends into space otherwise occupied by the Phe370 side chain. BDP5290 binding displaces the loop from Asn369 onwards and causes the Phe370 side chain to rotate by 120°. Considering that these residues are directly N-terminal of an extended loop that is disordered in all MRCKβ structures, and Phe370 itself appears to be somewhat flexible in the ADP complex based on electron density quality, it is plausible that the BDP5290-induced conformational change does not carry an extensive energetic penalty. At the same time, it is noteworthy that the corresponding region of ROCK1 appears completely ordered [19], thus the requirement of BDP5290 for conformational change may contribute to its MRCK selectivity over ROCK kinases. The pyrazole amide of BDP5290 displaces one of the three pocket waters, with the ligand carbonyl taking its place and accepting a hydrogen bond from one of the other two waters (Figure 3B). The chloropyrazole moiety occupies space not filled by either fasudil or TPCA-1 without making any obvious directional interactions with the protein, though it is possible that the positioning of the chlorine with its σ-hole pointing towards the Glu124 side chain and its partially negatively charged rim interacting with the side chain amine of Lys105 as well as the backbone NH of Asp218 makes a contribution to ligand affinity. The piperidine of BDP5290 (Figure 3B) occupies approximately the same space as the fasudil piperazine [11]. It packs against the amide plane between Arg84 and Gly85, but otherwise points towards solvent.

### MRCK inhibition in cells

**Figure 4:**
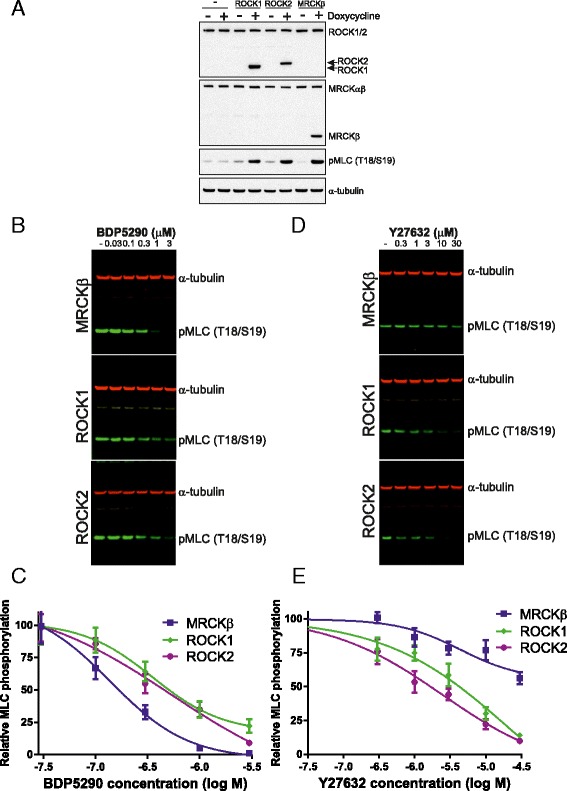
**Inhibition of kinase activity by BDP5290 in cells. (A)** MDA-MB-231 breast cancer cells expressing doxycycline inducible ROCK1, ROCK2 or MRCKβ kinase domains were established as indicated. Following treatment with doxycycline for 18 hours to induce expression, cell lysates were western blotted with antibodies as indicated. **(B)** Cells expressing doxycycline-induced MRCKβ, ROCK1 or ROCK2 kinases domains were treated with BDP5290 at indicated concentrations for 60 minutes prior to lysis and quantitative western blotting. **(C)** Inhibition of MLC phosphorylation by BDP5290 for each induced kinase domain. **(D)** Cells expressing doxycycline-induced MRCKβ, ROCK1 or ROCK2 kinases domains were treated with Y27632 at indicated concentrations for 60 minutes prior to lysis and quantitative western blotting. **(E)** Inhibition of MLC phosphorylation by Y27632 for each induced kinase domain. All results are shown as mean ± standard error of n = 4 independent replicates.

To determine whether the inhibitor BDP5290 would inhibit MRCK in cells, we established cell lines expressing doxycycline-inducible ROCK1, ROCK2 or MRCKβ kinases domains that led to increased pMLC following doxycycline treatment for 18 hours (Figure 4A). By treating cells in which ROCK1, ROCK2 or MRCKβ had been induced with doxycycline with varying concentrations of BDP5290 from 0 to 3 μM (Figure 4B), cell based EC_50_ values were determined to be 166 nM for MRCKβ, 501 nM for ROCK1 and 447 nM for ROCK2 (Figure 4C). Interestingly, 3 μM BDP5290 completely inhibited MLC phosphorylation induced by MRCKβ, but not by ROCK1 or ROCK2. For comparison, similar experiments were performed with the ROCK selective inhibitor Y27632 [20] with concentrations ranging from 0 to 30 μM (Figure 4D). Inhibition of MLC phosphorylation induced by ROCK1 had an EC_50_ value of 4.27 μM and for ROCK2 was 1.62 μM (Figure 4E). Although Y27632 had some effect on MRCKβ activity, inhibition was not greater than 50% at the highest 30 μM concentration. These results demonstrate that BDP5290 is a potent inhibitor of MLC phosphorylation in cells with selectivity for MRCK over ROCK1 or ROCK2.

**Figure 5:**
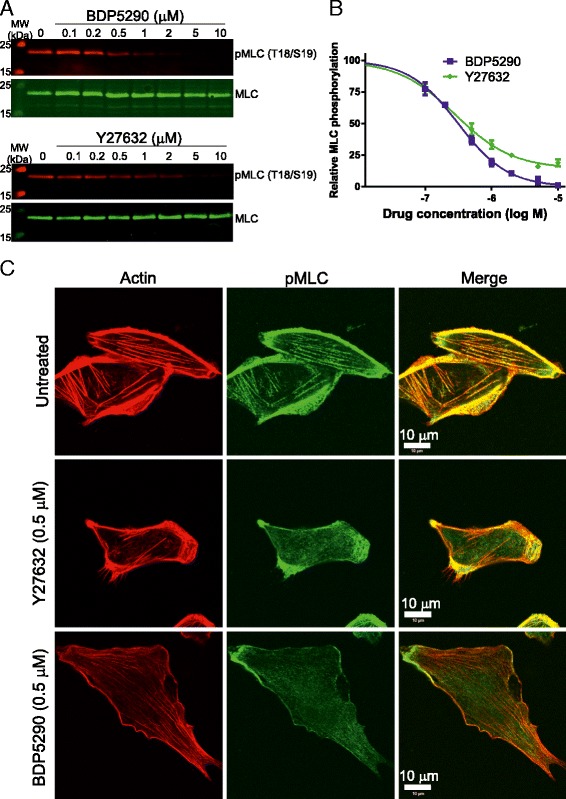
**Inhibition of myosin light chain phosphorylation in MDA-MB-231 breast cancer cells by BDP5290. (A)** MDA-MB-231 human breast cancer cells were treated with indicated concentrations of BDP5290 or Y27632 for 30 minutes prior to cell lysis and quantitative western blotting with indicated antibodies. **(B)** Inhibition of MLC phosphorylation by BDP5290 and Y27632. All results are shown as mean ± standard error of n = 3 independent replicates. **(C)** Immunofluorescence imaging of MDA-MB-231 cells that were untreated or treated with 0.5 μM Y27632 or BDP5290 for 30 minutes. After fixation and permeabilization, cells were stained with phalloidin to visualize filamentous actin structures and pMLC (S19) primary antibody as indicated.

To determine how inhibition of endogenous MRCK and ROCK affected pMLC levels, we treated parental MDA-MB-231 breast cancer cells with BDP5290 or Y27632 at varying concentrations (Figure 5A). BDP5290 had an EC_50_ of 316 nM while Y27632 was slightly less potent with an EC_50_ of 407 nM (Figure 5B). At higher concentrations, BDP5290 reduced pMLC to undetectable levels while Y27632 was unable to completely inhibit pMLC on western blots. Immunofluorescence microscopy of MDA-MB-231 cells revealed both cytoplasmic stress-fibre associated and cortical pMLC (Figure 5C). Treatment for 30 minutes with inhibitors at concentrations near their EC_50_ on Western blots showed that 0.5 μM Y27632 effectively reduced stress-fibre associated pMLC staining but had little effect on cortical pMLC, in agreement with previous reports showing that ROCK principally phosphorylates cytoplasmic MLC [10]. In contrast, application of 0.5 μM BDP5290 lessened both cytoplasmic and cortical pMLC levels, which is consistent with previous reports showing that an important site of MRCK function is at cortical cytoskeletal structures proximal to the plasma membrane [21,22].

### MRCK inhibition reduces tumour cell motility and invasion

**Figure 6:**
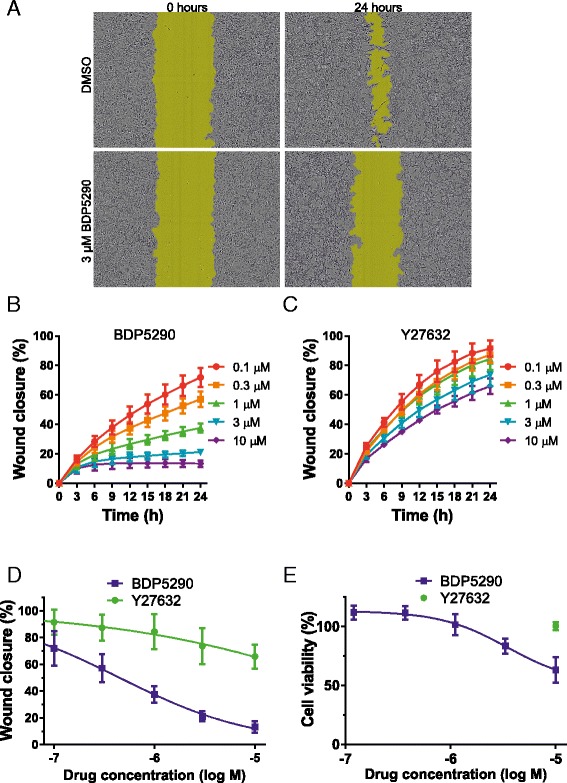
**Matrigel invasion of MDA-MB-231 cells is inhibited by BDP5290. (A)** MDA-MB-231 human breast cancer cells were plated in 96 well plates, then 24 hours later a ~800 μm scratch wound was created. Matrigel was overlayed for 1 hour, then images were acquired after 0 and 24 hours in the presence of DMSO vehicle or 3 μM BDP5290. **(B)** Relative wound closure of MDA-MB-231 cells imaged up to 24 hours at 3 hour intervals in the presence of indicated BDP5290 concentrations. **(C)** Relative wound closure of MDA-MB-231 cells imaged up to 24 hours at 3 hour intervals in the presence of indicated Y27632 concentrations. **(D)** Relative wound closure of MDA-MB-231 cells at 24 hours in the presence of indicated BDP5290 or Y27632 doses. All results are shown as mean ± standard error of n ≥ 4 independent replicates. **(E)** Alamar Blue metabolism of MDA MB 231 cells after 24 hours in the presence of DMSO vehicle, indicated concentrations of BDP5290 or 10 μM Y27632. Readings were compared to the Alamar Blue metabolism in DMSO treated cells that were used as the viability standard of 100% for each replicate experiment. All results are shown as mean ± standard error of 3 independent replicates.

We and others used siRNA to show that MRCK activity contributes to the ability of MDA-MB-231 human breast cancer cells to invade three dimensional Matrigel [6,11] and SCC12 human squamous cell carcinoma cells to invade collagen in an organotypic skin culture model of invasion [12]. Using a 96-well based Matrigel invasion assay and measurements determined with an Incucyte live content imaging instrument at time points up to 24 h (Figure 6A), we found that BDP5290 reduced MDA-MB-231 invasion at all tested concentrations starting from 0.1 μM, with virtually complete inhibition at 10 μM (Figure 6B). In contrast, Y27632 was dramatically less effective at inhibiting invasion at all concentrations, (Figure 6C). Comparing the dose–response relationship at 24 h after the start of the experiments, the EC_50_ for BDP5290 was 440 nM (Figure 6D), which was similar to the EC_50_ for inhibiting MLC phosphorylation (Figure 5B). However, Y27632 inhibition of invasion was not greater than 50% even at 30 μM (Figure 6D). To ensure that BDP5290 did not affect MDA MB 231 cell viability, a range of BDP5290 concentrations were tested for their effects on metabolic activity relative to DMSO vehicle using Alamar Blue [23]. After 24 hours in the presence of BDP5290 cell viability as measured by Alamar Blue metabolism was slightly reduced with an EC_50_ > 10 μM (Figure 6E). Wound closure was inhibited by > 60% at 1 μM BDP5290 (Figure 6D), a concentration that had no effect on cell viability (Figure 6E), indicating that the MRCK inhibitor can directly block cell motility independent of effects on cell proliferation. Treatment with 10 μM Y27632 had no effect on cell viability (Figure 6E) but inhibited wound closure by ~40%, indicating that ROCK inhibition also reduced cell motility directly. Given that both inhibitors had similar effects on total MLC phosphorylation (Figure 5A and B) but their effects on pMLC in different cellular compartments varied (Figure 5C), one possibility is that the phosphorylation of cortical MLC is an important contributor to cell motility and invasion. Previous studies found that polarized cell motility was dependent on recruitment of MRCK to the leading edge [22], where it promotes actin-myosin retrograde flow to generate tractive forces for cell movement [9]. One mechanism identified for this recruitment is the translocation of MRCKα associated with the PDK1 kinase that binds membrane phosphatidylinositol (3,4,5)-trisphosphate [24]. Blocking this translocation impairs the ability of MRCKα to promote lamellipodia retraction with consequent inhibition of directional migration. In addition, MRCK was found to be required for the assembly of matrix degrading complexes containing MT1-MMP [25] and promote cathepsin B expression [26] to permit cell invasion *via* matrix degradation. Therefore, BDP5290 may also affect matrix degradation as well as cell motility, resulting in significant inhibition of invasion.

**Figure 7:**
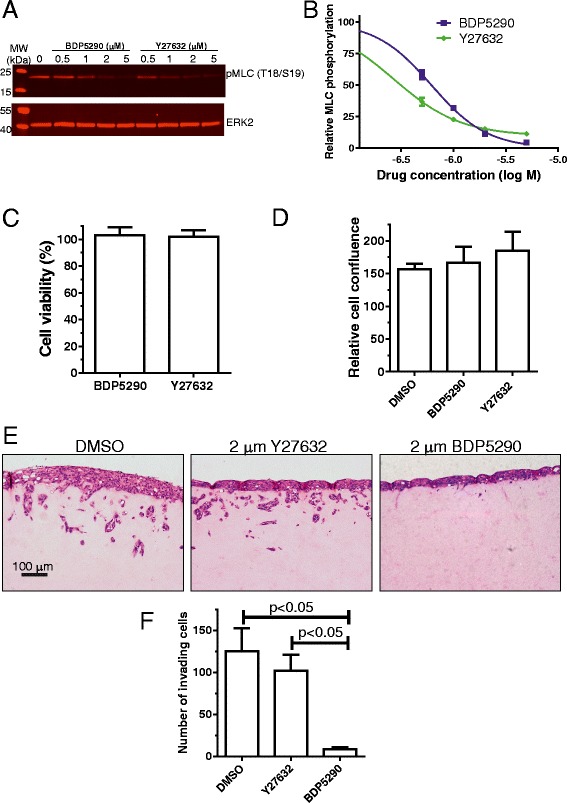
**Organotypic collagen invasion of SCC12 cells is inhibited by BDP5290.** **(A)** SCC12 human squamous cell carcinoma cells were treated with indicated concentrations of BDP5290 or Y27632 for 30 minutes prior to cell lysis and quantitative western blotting with indicated antibodies. **(B)** Inhibition of MLC phosphorylation by BDP5290 and Y27632. All results are shown as mean ± standard error of n = 3 independent replicates. **(C)** Alamar Blue metabolism of SCC12 cells after 24 hours in the presence of DMSO vehicle, 2 μM BDP5290 or 2 μM Y27632. Readings were compared to the Alamar Blue metabolism in DMSO treated cells that were used as the viability standard of 100% for each replicate experiment. **(D)** Percentage cell confluence of 35,000 cells (set to 100% at t = 0) plated in replicate wells of 96 well plates in the presence of DMSO vehicle, 2 μM BDP5290 or 2 μM Y27632 was determined after 21 hours by imaging with an Incucyte Zoom. **(E)** Example images of SCC12 cells invading collagen, that had been conditioned by cancer associated fibroblasts for five days prior to their removal, in the presence of DMSO vehicle 2 μM BDP5290 or 2 μM Y27632 for 10 days. **(F)** Number of invading cells was determined in 5 random fields for each condition for each separate replicate experiment. All results are shown as mean ± standard error of n ≥ 3 independent replicates.

In an organotypic invasion model, the ability of SCC12 cells to collectively invade through physical tracks made by carcinoma associated fibroblasts (CAF) in three-dimensional collagen matrices was found to be dependent on MRCK, and not ROCK [12]. When the effects of varying doses of BDP5290 and Y27632 on phosphorylated MLC in SCC12 cells were compared (Figure 7A), both inhibitors were found to have similar potencies, although Y27632 showed residual phosphorylated MLC at higher doses (Figure 7B) as was the case in MDA-MB-231 breast cancer cells (Figure 5B). To ensure that MRCK or ROCK inhibition did not affect SCC12 cell viability, DMSO vehicle, 2 μM BDP5290 or 2 μM Y27632 were tested for their effects on metabolic activity using Alamar Blue [23]. After 24 hours in the presence of 2 μM BDP5290 or 2 μM Y27632, cell viability as measured by Alamar Blue metabolism did not differ from DMSO standard values (Figure 7C). In addition, when equal numbers of cells were plated in DMSO vehicle, 2 μM BDP5290 or 2 μM Y27632, cell confluence as determined by Incucyte image analysis did not significantly differ between treatments (Figure 7D). Therefore, neither drug affected SCC12 cell viability or proliferation. Following CAF-conditioning of the collagen matrix for five days and subsequent removal of the cells with puromycin, SCC12 cells were layered on the surface of the protein matrix, the collagen plug was raised above the air-medium interface and carcinoma cell invasion occurred over ten days in the presence of DMSO vehicle, 2 μM Y27632 or 2 μM BDP5290 as indicated (Figure 7E). Consistent with previous observations [12], ROCK inhibition did not significantly affect SCC12 invasion into the three dimensional collagen matrix (Figures 7E and F). However, 2 μM BDP5290 had a profound effect on SCC12 invasion (Figures 7E and F). These data indicate that MRCK inhibition induces phenotypic responses consistent with the effects previously observed by siRNA-mediated knockdown of MRCK [12], which validates its use as a tool compound to explore the contributions of MRCK to tumour cell invasion and metastasis.

## Conclusions

In this study we report the discovery of BDP5290, a potent small molecule inhibitor with marked selectivity for the MRCK kinases. Although the MRCK and ROCK kinase domains are very similar, X-ray crystallography revealed some features that contribute to inhibitor selectivity. An intriguing feature of the MRCKβ · BDP5290 complex is the presence of the two pocket waters that are enveloped by the protein and ligand (Figure 3B). These waters may contribute to MRCK selectivity; the replacement of Leu128 of MRCKβ with a methionine in ROCK1 likely means that the outer water (on the left side in Figure 3B) is absent in ROCK1. This is supported by the available ROCK1 crystal structures, all of which show either only the inner water or no waters at all in this pocket (the latter presumably due to the low resolution of the structures). As a consequence, a ligand that interacts favourably with the outer water (*e.g.* BDP5290) should favour binding to MRCKβ over ROCK1. On the other hand, the ROCK selective inhibitor fasudil displaces the outer water without replacing its interactions [11], making it a poor MRCKβ ligand while not affecting ROCK1 binding. Therefore, it may be possible to increase ligand potency by improving interactions with these solvent molecules or by replacing them appropriately.

In comparison to the widely-used ROCK selective inhibitor Y27632, BDP5290 was more effective at reducing MLC phosphorylation at cortical actin-myosin structures (Figure 5C), consistent with the reported site of MRCK action being proximal to plasma membranes. In two models of tumour cell invasion, BDP5290 was much more effective than Y27632 at reducing invasiveness (Figures 6 and 7). It had been previously reported that invasion by MDA-MB-231 breast cancer cells was partially inhibited by ROCK or MRCK inhibition/knockdown, but that full block of invasion required inhibition of both pathways [6,11]. One possible interpretation of these results is that the two kinase families act in concert through independent and non-compensatory mechanisms. Given that BDP5290 is MRCK selective but with some inhibitory effect on ROCK activity while Y27632 is predominantly ROCK selective, the greater effectiveness of BDP5290 at blocking MDA-MB-231 invasion may be the result of this dual kinase targeting. The observation that BDP5290 completely blocks MLC phosphorylation is consistent with it being a dual MRCK/ROCK inhibitor, while the residual MLC phosphorylation in Y27632 treated cells may reflect its inability to effectively inhibit MRCK in cells. Given that BDP5290 and Y27632 were similar in their potencies for inhibiting MLC phosphorylation and yet were markedly different in their ability to block tumour cell invasion, an alternative possibility is that inhibition of cortical MLC phosphorylation is of greater importance than cytoplasmic MLC. In the case of SCC12 invasion, the absolute dependence on MRCK was linked to MLC phosphorylation around the periphery of the cell collective [12]. It is also possible that the actions of MRCK proximal to the plasma membrane lead to phosphorylation of additional substrates that contribute to invasion, which are less likely to be regulated by more cytoplasmic ROCK proteins. For example, MRCK was found to be important for assembly of matrix degrading complexes containing the transmembrane MT1-MMP metalloproteinase, which may reflect its actions near the plasma membrane [25].

Proteins that might contribute to cancer are often identified on the basis of changes in gene expression or mutations. If MRCK were important in promoting tumour invasion and metastasis, it would be predicted that signalling through this pathway would be increased in metastatic tumour cells. *MRCKα* (designated PK428 in this study) expression was found to be elevated in breast tumours, being part of a “poor prognosis” gene expression signature with increased incidence of distant metastases in less than 5 years [14]. Consistent with these observations, increased *MRCKα* gene copy number was detected in 542 of 852 (64%) breast cancer samples at the Wellcome Trust Sanger Institute Cancer Genome Project (https://www.sanger.ac.uk/research/projects/cancergenome/). In addition, the activity of the upstream regulatory Cdc42 protein might be elevated in tumours by a number of possible mechanisms including; increased expression, changed activity of positive or negative regulatory proteins, or by increased stimulation from extracellular signals in the tumour microenvironment. Rather than relying on expression levels as surrogate markers for activity, reagents that reported on MRCK activity would be valuable to identify cancers in which this signalling pathway is activated. In addition, these reagents would have the potential to be useful as pharmacodynamic markers of *in vivo* drug effects. This approach has been used for ROCK1 and ROCK2, where autophosphorylation on Ser1333 and Ser1366 respectively were determined to reflect activity [27,28]. In the case of ROCK2, phospho-selective antibodies against this Ser1366 post-translational modification were used to stain breast cancer clinical samples to detect ROCK2 activation.

Despite the apparent importance of MRCK as a regulator of actomyosin contractility, there is significantly less known about them than for ROCK. One important reason for the breadth of knowledge about ROCK function is because of the discovery of ROCK selective inhibitors such as Y27632, which was first published in 1997 [20]. The ready availability of Y27632 and subsequently improved inhibitors has facilitated research into ROCK in numerous disease indications. To date, few inhibitors that affect MRCK activity have been reported. Chelerythrine was first identified as a Protein Kinase C inhibitor with *in vitro* IC_50_ of 660 nM [29], and subsequently was reported to inhibit MRCKα with an *in vitro* IC_50_ of 1.77 μM [30]. MRCKα inhibition by chelerythrine was determined to be through a non-ATP-competitive mechanism, but the site of ligand binding has not been determined. Poor selectivity makes chelerythrine difficult to use for cell-based experiments to evaluate MRCK function, additional reported off-target effects include inhibition of acetylcholinesterases [31], reactive oxygen species generation [32] and DNA intercalation [33]. The discovery of inhibitors that potently inhibit MRCK activity will allow for functional studies to be undertaken, and the potential therapeutic value of MRCK targeting for disease treatments to be determined.

## Methods

### Kinase assays

MRCKα, MRCKβ, ROCK1 and ROCK2 assays were performed using an IMAP fluorescence polarization assay format (Molecular Devices Inc.). 8–12 nM of each kinase (Life Technologies) was incubated for 60 min at room temperature with 100 nM FAM-S6-ribosomal protein derived peptide (synthesized by Alta Biosciences, University of Birmingham UK) in the presence of 1 μM ATP and 0.5 mM MgCl_2_ in 20 mM Tris buffer (pH 7.4) containing 0.01% Tween-20 and 1 mM DTT (MRCKα and β); or 1 μM ATP, 10 mM MgCl_2_ in 20 mM Tris buffer (pH 7.5) containing 0.25 mM EGTA 0.01% Triton X-100 and 1 mM DTT (ROCK1 and ROCK2). Typically, dose response analyses were performed over concentration ranges from 0.005 - 100 μM. Reactions were stopped by adding 2 assay volumes of 0.25% (v/v) IMAP binding reagent in 1× IMAP binding buffer A (Molecular Devices). After 30 min incubation to allow binding reagent to bind phosphorylated peptide, fluorescence polarization was measured on a Tecan Saphire^2^ plate reader at excitation (470 nm) and emission (530 nm) wavelengths. Inhibition was calculated using no inhibitor and no enzyme controls as 0 and 100% inhibition, respectively. Kinase selectivity profiling was performed by Eurofins with 10 μM ATP and 10 μM BDP5290.

### Protein expression and crystallization

The kinase domain of human MRCKβ (residues 2–417) was expressed and crystallized as described previously [11]. Crystals of the MRCKβ BDP5290 complex were obtained by addition of 0.15 μl of compound stock (10 mM in DMSO) to a 1.8 μl drop containing ligand-free crystals for 24 h before cryoprotection in mother liquor supplemented with 20% ethylene glycol and data collection.

### Data collection, processing, structure solution and refinement

Data for both ADP-bound protein and the BDP5290 complex were collected at beamline I24 of Diamond Light Source (Didcot, UK). They were processed and scaled using XDS [34] to a resolution of ≈ 1.70 Å. Phases were obtained by molecular replacement using MOLREP [35] with a protein monomer from the MRCKβ · fasudil complex (PDB ID 3TKU; [11]) as the search model. Refinement proceeded through cycles of model building in Coot [36] and refinement with PHENIX (ADP complex; [37]) or REFMAC (BDP5290 complex; [38]); restraints and starting coordinates for BDP5290 were generated using PRODRG [39]. The quality of the final structures was assessed using Coot and PROCHECK [40]. Data collection and refinement statistics are shown in Table 2.

**Table 2:** **Data collection and refinement statistics**

	**ADP complex**	**BDP5290 complex**
Wavelength (Å)	0.98	0.97
Space group	C2	C2
Cell dimensions	*a* = 114.2 Å; *b* = 44.0 Å; *c* = 91.3 Å; β = 107.4°	*a* = 115.0 Å; *b* = 44.0 Å; *c* = 91.1 Å; β = 107.3°
Resolution (Å)	26.50–1.73 (1.78–1.73)	40.00–1.71 (1.75–1.71)
Total reflections	143025 (9319)	197075 (15104)
Unique reflections	44725 (3283)	046470 (3438)
Completeness (%)	98.8 (99.2)	98.0 (98.7)
Redundancy	3.2 (2.8)	4.2 (4.4)
*I*/σ(*I*)	11.5 (2.2)	12.7 (2.1)
*R*_merge_	0.047 (0.554)	0.066 (0.571)
*R*_work_/*R*_free_	0.196/0.243	0.205/0.236
r.m.s.d. from ideal geometry:		
Bond lengths (Å)	0.013	0.017
Bond angles (°)	1.70	1.69
Average *B* values:		
Overall (Å^2^)	30.4	29.0
Protein (Å^2^)	30.0	28.8
Solvent (Å^2^)	35.7	32.2
Ligand (Å^2^)	36.2	23.3
PDB ID	4UAK	4UAL

Values in parentheses pertain to the highest resolution shell of ≈ 0.05 Å.

### Cell culture

MDA-MB-231 D3H2LN-Luc cells (Caliper LifeScience) were grown in MEM/EBSS (Hyclone) supplemented with 10% fetal bovine serum (FBS) (Gibco), 1 mM Sodium Pyruvate (Sigma) 2 mM L-glutamine (Gibco), 1× MEM non-essential amino acids (Gibco) plus penicillin-streptomycin (10 Units/ml and 10 μg/ml respectively) at 37°C in 5% CO_2_ in a humidified incubator. MDA-MB-231 D3H2LN-Luc TetOn MRCKβ, ROCK1 and ROCK2 selective cell lines were grown in the same media with 10% FBS replaced with 10% Tet System Approved FBS (Clontech).

HN-CAF cells (a gift from Erik Sahai, Cancer Research UK London Institute) were grown in DMEM (Gibco, 21969–035) with 10% fetal bovine serum (FBS) (Gibco, 10270), 2 mM L-glutamine (Gibco, 25030–032) at 37°C in 5% CO_2_ in a humidified incubator. SCC12 cells (a gift from Erik Sahai) were grown in 2/3 DMEM, 1/3 Ham’s nutrient F12 (Gibco, 21765–029), 2 mM L-glutamine, 10%FBS, 5 μg insulin (Sigma, I0516), 10 ng/mL EGF (Sigma, E9644) and 0.5 μg/ml hydrocortisone (Sigma, H-0135).

### Tet-inducible cell line generation

MDA-MB-231 D3H2LN-Luc cells stably expressing the Tet-On 3G (Tet3G) transactivator (Clontech) were generated according to the manufacturer’s instructions. Briefly, MDA-MB-231 D3H2LN-Luc cells were transfected with pCMV-Tet3G (Clontech) using Xfect transfection reagent (Clontech) and selected with G418 (600 μg/ml). G418-resistant colonies were isolated, expanded, and Tet3G transactivator expression determined by western blot analysis using the TetR antibody (Clontech). Clonal cell lines expressing the Tet3G protein were transfected with pTRE3G-Luc reporter construct, treated with and without doxycycline (1 μg/ml), and luciferase activity determined. The clone exhibiting the lowest basal activity and greatest doxycycline induction was used to generate the Tet-On inducible cell lines described below.

The kinase domains of MRCKβ (1–473), ROCK1 (1–535) or ROCK2 (1–532) were gene synthesized (Genscript) and subcloned into pTRE3G (Clontech). MDA-MB-231 D3H2LN-Luc Tet3G cells were co-transfected with pTRE3G-MRCKβ (1–473), pTRE3G-ROCK1 (1–535) or pTRE3G-ROCK2 (1–532), together with a linear Pur^r^-selection marker using Xfect transfection reagent according to manufacturer’s instructions. Following selection in puromycin (0.5 μg/ml), single colonies were isolated and expanded. Each clone was treated with or without doxycycline (1 μg/ml) for 24 hours and kinase domain expression and activity determined by western blotting. For each Tet-inducible kinase, clones were chosen which showed robust inductions of kinase domain expression together with the highest-fold induction of phospho-MLC2 (Thr18/Ser19) expression in response to doxycycline treatment.

### Cell extraction and immunoblot analysis

Cell lysates were prepared as described previously [41]. For pMLC western blots, cells were lysed in a 1% (w/v) SDS, 50 mM Tris pH 7.5 buffer and lysates were passed through QIAshredder columns (Qiagen, 79654). Alternatively, for pMLC western blots lysates were prepared in lysis buffer with 50 mM Tris (pH7.5), 0.5% (w/v) SDS, supplemented with Complete Protease and PhosSTOP inhibitors (Roche). Whole cell lysates were separated by SDS-PAGE, transferred to Protran nitrocellulose membranes (Whatman), probed with primary antibodies and appropriate IR dye-conjugated secondary antibodies. Blots were visualized using a Licor Odyssey according to manufacturer’s instructions. Antibodies used in this study were: MRCKα (611584) from BD Transduction Laboratories; MRCKβ (H00009578-A01) from Abnova; MRCKα/β (MANDM1 6G8) from Glenn Morris (Centre for Inherited Neuromuscular Disease, Oswestry UK) [42,43]; MRCL3/MRLC2/MYL9 (sc-28329) from Santa Cruz Biotechnology; pMLC2 Thr18/Ser19 (3674) from Cell Signaling Technology. ROCK1/2 (07–1458) from Millipore; TetR (Clone 9G9; 631132) from Clontech; α-Tubulin (TU-02; sc-8035) from Santa Cruz Biotechnology; ERK2 from Chris Marshall (Institute of Cancer Research, London UK). Secondary antibodies used were: goat anti-mouse IgG Dylight 800 from Thermo Scientific (35521), goat anti rabbit IgG AlexaFluor 680 from Invitrogen (A12076), goat anti-rabbit IgG IR Dye 800 CW (926–32211) and goat anti-mouse IgG IR Dye 680 (926–68020) were from LI-COR Biosciences.

### Immunofluorescence

MDA-MB 231 cells were grown on glass coverslips in 24 well plates. Cells were treated for 30 minutes with either DMSO vehicle, 0.5 μM Y27632 (Calbiochem, #688000) or 0.5 μM BDP5290. Cells were then washed with PBS, fixed in 4% para-formaldehyde (PFA; EMS #15710) in PBS for 15 minutes, washed twice in PBS, permeabilized with 0.5% Triton X100 (Thermo Scientific, #28314) in PBS for 15 minutes and washed twice in PBS. After blocking for 30 minutes in PBS with 1% BSA, fixed cells were incubated with pMLC2 Ser19 antibody (Cell Signaling Technology, #3675) for 1 hour at room temperature. Cells were washed three times in PBS with 1% BSA, and incubated in the dark with secondary antibody anti-mouse Alexa Fluor 488 (Invitrogen, A11029) and Texas Red Phalloidin (Invitrogen, T7471) for 1 hour at room temperature. Cells were washed three times in PBS with 1% BSA, once in PBS and once in water. Coverslips were mounted with Vectashield (Vector). Slides were visualized with a Zeiss 710 confocal microscope. Post-imaging linear adjustments for brightness and contrast were made uniformly for all image panels to improve the clarity and discernibility of subcellular structures.

### Matrigel invasion assays

MDA-MB-231 cells were plated at 30,000 cells per well in Image Lock 96 well plates (Essen BioScience) in 100 μL complete DMEM medium and after allowing cells to settle for 20 min were incubated overnight at 37°C in 5% CO_2_ in a humidified incubator. Matrigel (BD Biosciences) was thawed overnight at 4°C, diluted 1:1 with PBS and 100 μL pipetted over cells that had been scratched with the WoundMaker tool (Essen BioScience). Plates were incubated for 1 h, then appropriately diluted drugs added in 100 μL media to achieve final concentrations. Plates were scanned with an Incucyte Zoom (Essen BioSciences) according to manufacturer’s instructions at 3 hour intervals. Cell viability was determined with AlamarBlue (Invitrogen, DAL 1025), which was added to the medium and cells cultured for 6 hours. Absorbances at 570 nm and at 600 nm were measured to assess metabolic activity.

### Organotypic invasion assays

SCC12 invasion assays were performed essentially as described in [12]. HN-CAF cells were grown for 5 days in a Matrigel-collagen gel, covered with DMEM + 10%FBS medium, and allowed to remodel the matrix. The gel consisted of: 280 μL of DMEM + 10% FBS, 100 μL FBS, 20 μL 1 M Hepes pH 7.5 and approximately 200 μL of Matrigel (BD Biosciences, 354234), 400 μL of collagen type 1 (BD Biosciences, 354249) and 5×10^5^ HN-CAF cells. Puromycin was subsequently added to the medium at 10 μg/ml for 3 days to remove HN-CAF cells. The gels were washed in SCC medium 3 times for 2 hours and SCC12 cells were plated on the surface of the gel and left to adhere overnight. The gels were placed on nylon filters supported by metal grids and cultured for 10 days at the interface with SCC medium and either DMSO vehicle, 2 μM Y27632 or 2 μM BDP5290. The gels were fixed overnight in 4% PFA and 1% glutaraldehyde in PBS, washed in PBS and processed for histology.

### Alamar Blue and confluence analysis

MDA MB 231 or SCC12 cells were plated in a 96 well plate and cultured for 24 hours. Cells were then cultured for 24 hours in SCC12 medium with DMSO vehicle, 2 μM Y27632 or indicated concentrations of BDP5290 in an IncuCyte ZOOM (Essen Bioscience). Pictures were taken every 3 hours and confluence was measured using the IncuCyte analysis software. AlamarBlue (Invitrogen, DAL 1025) was added to the medium and the cells were cultured for an additional day. Absorbances at 570 nm and at 600 nm were measured to assess cell health.

## Abbreviations

ADP: Adenosine diphosphate
ANOVA: Analysis of variance
ATP: Adenosine triphosphate
CAF: Cancer associated fibroblast
DMEM: Dulbecco’s modified Eagle’s medium
DTT: Dithiothreitol
EC_50_: Half maximal effective concentration
HN-CAF: Head and neck cancer associated fibroblast
MLC: Myosin II light chain
MRCK: Myotonic dystrophy kinase-related CDC42-binding kinase
pMLC: Phosphorylated myosin II light chain
RMSD: Root mean square deviation
ROCK: Rho-associated coiled-coil kinase
SAR: Structure-activity relationship
SCC: Squamous cell carcinoma
SDS-PAGE: Sodium dodecyl sulphate polyacrylamide gel electrophoresis
